# Anti-Herpes Simplex Viruses activity of *Camellia sinensis*, member of the family Theaceae (green tea)

**DOI:** 10.1186/1471-2334-14-S3-P47

**Published:** 2014-05-27

**Authors:** Govindharaj Deepika, Hariharan Durgadevi, Rohan Narayan, Munisamy Sudanthira, Elanchezhiyan Manickan

**Affiliations:** 1 Department of Microbiology, Dr. ALM PG IBMS, University of Madras, Chennai, India

## Background

Medicinal plants were shown to possess several bioactivities of which *Camellia sinensis*, a popularly used refreshing beverage has been reported to have immune modulating and anti viral activities. From our lab we had already shown that *C. sinensis* extracts possessed anti-HIV and anti-HBV activities. In the current study we evaluated its anti-HSV activities.

## Methods

Aqueous and methanolic extracts of *C. sinensis* were tested for their anti-HSV property by CPE reduction assay on Vero cell monolayers. Cytotoxicity of the extract was measured by MTT assay.

## Results

Experiments showed that 12 µg/mL concentration of *C. sinensis* completely inhibited HSV-1 and 2 and the drug was found to be non toxic up to 10 mg/mL.

## Conclusion

The study showed that *C.sinesis* extracts and EGCG compound are potent inhibitor of both HSV-1 and HSV-2 infection and it was found to be relatively non toxic.

